# Editorial: Remote work burnout during the COVID-19 pandemic

**DOI:** 10.3389/fpsyg.2024.1389984

**Published:** 2024-06-06

**Authors:** Tomas Kliestik, Katarina Valaskova, Dan-Cristian Dabija

**Affiliations:** ^1^Department of Economics, Faculty of Operation and Economics of Transport and Communications, University of Zilina, Zilina, Slovakia; ^2^Department of Marketing, Faculty of Economics and Business Administration, Babeş-Bolyai University Cluj-Napoca, Cluj-Napoca, Romania

**Keywords:** editorial, burnout, COVID-19 pandemic, remote work burnout, remote work

The general stress of everyday life, together with increasingly complex and various responsibilities, the need for office interactions and engagement in unprecedented situations, as well as management and company pressures to identify new and innovative solutions to different challenges, tasks, and strategies led at alarming rates to burnout (Raišiene et al., 2023; Berglund et al., 2024). Burnout has thus become a scourge found particularly among employees working full-time in the office, as a result of increased levels of stress, anxiety, and isolation, along with reduced break time and time off, in which they can relax and replenish their strength (Ferreira et al., 2024).

Employees experienced semblances of burnout and strong emotional distress, especially during the recent COVID-19 pandemic, which radically changed how they carried out everyday tasks and responsibilities in the workplace. Due to the fact that employees suddenly had to give up their daily lives, which meant commuting from home to work (Garcia et al., 2024), in-person peer interaction (Lal et al., 2023), third-party socialization, and tackling various new situations meant that the pandemic had generated a stress disorder (Zhen et al., 2023), distress, and prolonged emotional stress. Many employees who felt trapped in their homes rapidly became exhausted and overwhelmed by their duties, tasks, work responsibilities, and job demands (Jamal et al., 2024), which led to a drastic decrease in productivity, work output, and efficiency. Their workloads were not lessened; on the contrary, the workloads were at times increased, so that employees had to rapidly adapt to new situations and learn new tech skills to get access to company servers from their homes (Ingusci et al., 2021), to communicate solely virtually with their coworkers, clients, vendors, and stakeholders. Faced with such unexpected challenges, longer working hours, and excessive job expectations, employees felt signs of mental overload, strain, exhaustion, overwork, mental stress, anxiety, and depression, handling work with difficulty and decreasing work performance and task performance (Kumar et al., 2021). There is a chance that these negative aspects generate job dissatisfaction and psychological and health issues, turnover intention, and counterproductive work behavior (Nemţeanu et al., 2021).

From an organizational standpoint, it is essential to recognize ahead of time the stress-inducing situations leading to burnout and counterproductive work behavior in order to avoid unpleasant situations (Ali Awad and Mohamed El Sayed, 2023). By ensuring psychological wellbeing, a proper work-life balance, and employee support, work quality can be increased, and burnout can be avoided (Palma, 2021). Proper time management, mood tracker apps, and burnout assessment tools are essential in this context, which companies and organizations can implement to tackle this phenomenon (Adam et al., 2023).

The first article by Costin et al. delves into reviewing remote work burnout, professional job stress, and employee emotional exhaustion during the COVID-19 pandemic. The mini-review employed an assessment of the literature with the help of some methodological quality assessment tools, like the Methodological Quality of Systematic Reviews (AMSTAR), the Appraisal tool for Cross-Sectional Studies (AXIS), the Mixed Methods Appraisal Tool (MMAT), and the Systematic Review Data Repository (SRDR); data visualization was carried out with VOSviewer and Dimensions. After carefully visualizing and analyzing 44 up-to-date articles, the article provides a comprehensive overview of the impact of remote work on employee wellbeing during the COVID-19 pandemic, emphasizing the role that organizational management plays in understanding and supporting their employees. The article also highlights the necessity of proper control over scheduling in reducing psychological burnout and occupational stress while addressing the challenges and potential mitigating factors associated with remote work. The authors conclude that in order to deal with burnout and reduce their occupational stress, employees must benefit from organizational support systems and remote work adaptation.

The second article by Delaney et al. analyses mothers' experiences through the “mental lens” of working remotely from home during the recent COVID-19 pandemic. The research is based on data collected through interviews within a pan-European Horizon 2020 project; the analysis is qualitative in nature. The sudden transition from face-to-face to remote work during the recent COVID-19 pandemic led to blurred boundaries between work, care, and domestic labor for working mothers. That triggered a feeling that their home was “invaded” by work tasks and obligations, but also that part of the tasks remained unpaid and conflicted with their domestic responsibilities. The research illustrates the multi-layered experiences of working mothers and their perspectives toward paid and unpaid labor in relation to the mental loads with which they had to confront during the COVID-19 pandemic. Those lessons are of great importance and relevance in the post-pandemic context.

The third article by Felix et al. deals with individuals' responses to threats regarding remote worker identities but also the factors that generate such threats. Based on a grounded theory approach, the authors implement exploratory research, collecting 71 interviews from persons who chose to work remotely during the recent pandemic. The literature argues the main relevant concepts of the research, while in the practical part, the authors propose a model of responses to threats to remote workers identities, based identity-restructuring responses, identity-preserving responses, and paradoxical identity work responses. The study provides relevant insights into how remote workers were able to respond to negative stigma and theorizes the conditions under which each of the three responses might occur.

The last article by Ornaghi et al. enriches literature with original research on the role of teachers' socio-emotional competence in reducing burnout through increased work engagement. In this regard, the authors extract a conceptual model from the theory that emphasizes the relationships between perceived stress, teacher burnout, work engagement, and socio-emotional competence. Data was collected through quantitative research from 276 Italian in-service teachers, and the data was further investigated through structural equation modeling in AMOS. It was shown that perceived stress significantly impacts teachers' burnout, having both direct and indirect effects based on teachers' socio-emotional competencies and depending on their work engagement. From a managerial perspective, interventions should focus on reducing stress among teachers, fostering socio-emotional competencies, enhancing work engagement, and addressing age-related factors to promote teacher wellbeing and job performance.

Understanding the factors leading to symptoms of burnout among employees is and will be a great challenge for organizations that need to identify proper prevention measures, ensure appropriate wellbeing, and develop long-term mental stability among their employees.

## Author contributions

TK: Resources, Validation, Visualization, Writing – review & editing. KV: Resources, Validation, Visualization, Writing – review & editing. D-CD: Conceptualization, Investigation, Resources, Supervision, Validation, Visualization, Writing – original draft, Writing – review & editing.
